# BigMouth: development and maintenance of a successful dental data repository

**DOI:** 10.1093/jamia/ocac001

**Published:** 2022-01-23

**Authors:** Muhammad F Walji, Heiko Spallek, Krishna Kumar Kookal, Jane Barrow, Britta Magnuson, Tamanna Tiwari, Udochukwu Oyoyo, Michael Brandt, Brian J Howe, Gary C Anderson, Joel M White, Elsbeth Kalenderian

**Affiliations:** 1 Department of Diagnostics and Biomedical Sciences. School of Dentistry, University of Texas Health Science Center at Houston, Houston, Texas, USA; 2 Faculty of Dentistry. The University of Sydney, Sydney, Australia; 3 Office of Global and Community Health. Harvard School of Dental Medicine, Boston, Massachusetts, USA; 4 Department of Diagnostic Sciences. Tufts School of Dental Medicine, Boston, Massachusetts, USA; 5 Department of Community Dentistry & Population Health. University of Colorado School of Dental Medicine, Aurora, Colorado, USA; 6 Office of Dental Education Services. Loma Linda University School of Dentistry, Loma Linda, California, USA; 7 Office of Information Resources. University of Buffalo School of Dental Medicine, Buffalo, New York, USA; 8 Department of Family Dentistry. University of Iowa College of Dentistry and Dental Clinics, Iowa City, Iowa, USA; 9 Department of Developmental and Surgical Sciences. University of Minnesota School of Dentistry, Minneapolis, Minnesota, USA; 10 Department of Preventive and Restorative Dental Science. School of Dentistry, University of California at San Francisco, San Francisco, California, USA; 11 Department of Dental Management Sciences. School of Dentistry, University of Pretoria, Pretoria, South Africa

**Keywords:** dentistry, Research Patient Data Repositories, learning healthcare system, data governance

## Abstract

Few clinical datasets exist in dentistry to conduct secondary research. Hence, a novel dental data repository called BigMouth was developed, which has grown to include 11 academic institutions contributing Electronic Health Record data on over 4.5 million patients. The primary purpose for BigMouth is to serve as a high-quality resource for rapidly conducting oral health-related research. BigMouth allows for assessing the oral health status of a diverse US patient population; provides rationale and evidence for new oral health care delivery modes; and embraces the specific oral health research education mission. A data governance framework that encouraged data sharing while controlling contributed data was initially developed. This transformed over time into a mature framework, including a fee schedule for data requests and allowing access to researchers from noncontributing institutions. Adoption of BigMouth helps to foster new collaborations between clinical, epidemiological, statistical, and informatics experts and provides an additional venue for professional development.

## INTRODUCTION

**Figure 1.**
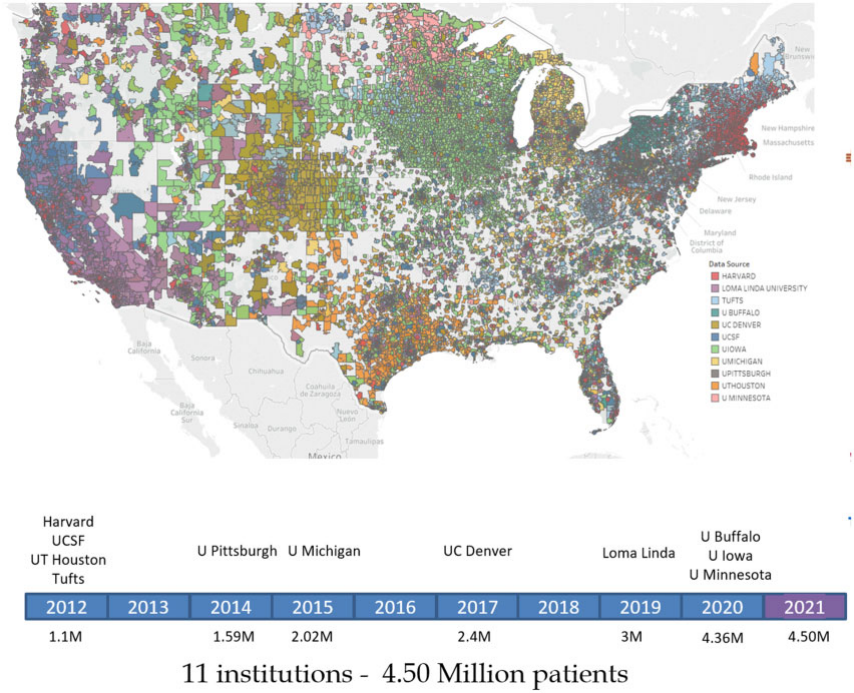
Timeline showing the year institutions began contributing to BigMouth, and current geographic coverage of patients (by zip code).

Each year in the United States, over 195 000 dental practitioners provide care to more than 127 million patients.^1^^,^^2^ Despite significant advances over time, researchers have had limited access to oral health datasets. While different Research Patient Data Repositories (RPDRs) exist, they rarely contain information on oral health that is associated with chronic conditions.^3^ To answer critical oral health-related research questions, investigators often rely on small local datasets, which are difficult to generalize. Alternatively, data are sometimes obtained from third-party payers (eg, dental insurance companies). The usefulness of these data may be limited as they are focused primarily on billed services, and many patients self-pay for dental care without involving a third party. The National Health and Nutritional Examination Survey^4^ and Behavioral Risk Factor Surveillance System^5^ are large oral health datasets, providing a view of the population’s dental status but contain limited information on dental diagnoses and actual treatments received. Linked datasets from medical and dental Electronic Health Records (EHRs) are also sorely lacking, impairing the ability to investigate relationships between oral health and general health.^6^^,^^7^ Recognizing this conundrum, we developed a centralized dental data repository using the i2b2 platform,^8^ called BigMouth.^9^ BigMouth was successfully launched in August 2012 with data on 1.1 million patients derived from dental EHRs of 4 dental schools—all members of the Consortium of Oral Health Research and Informatics (COHRI).^10^ Less than a decade later, BigMouth has grown into a formidable dental RPDR with 11 academic dental institutions contributing data on over 4.507 million patients (see Table 1) with diverse geographic coverage (see Figure 1).

**Table 1.** BigMouth data elements by contributing institutions/site

Data	Demographics	Diagnoses	Forms	Insurance	Odontogram	Periodontal Charts	Practice	Medications	Procedures
Site									
**UT Houston**	430 189	106 723	160 211	59 992	397 847	53 500	239 172	50 626	234 482
**UCSF**	993 959	261 980	121 888	126 537	844 575	79 931	571 218	42 558	635 216
**HSDM**	97 838	28 440	43 687	26 544	88 675	21 403	53 441	15 297	55 824
**Tufts**	425 100	0	127 228	122 673	379 490	83 898	241 787	56 502	245 925
**U Pitt**	291 648	0	103 591	71 319	256 299	30 856	115 668	36 328	117 202
**U Michigan**	484 781	33 780	133 216	227 235	420 802	61 358	307 904	46 009	308 348
**UC Denver**	167 180	14 714	48 986	44 644	142 594	34 117	99 143	41 096	105 688
**Loma Linda**	482 526	22 961	195 839	115 082	393 961	67 686	207 089	32 584	210 171
**U Buffalo**	231 398	22 741	21 317	9903	217 777	9822	23 812	12 021	25 065
**U Iowa**	801 739	898	464 412	325 697	665 953	53 844	518 717	142 689	512 259
**U Minnesota**	101 274	3689	101 274	77 535	101 274	46 393	95 410	49 836	97 019
**Total patients**	4 507 632	**495** **926**	1 478 902	**1** **207** **161**	**3** **909** **247**	**542** **808**	**2** **473** **361**	**525** **546**	**2** **547** **199**

## MAIN PURPOSE OF BigMouth

The objectives of BigMouth include informing the feasibility of research studies, executing informatic, population health, and observational studies, supporting quality improvement efforts, participation in data-driven research networks, and identifying clinical trial cohorts for recruitment. However, as a dental RPDR, BigMouth also fulfills several other purposes.

### Assessment of oral health status quo

BigMouth’s data on 4.5 million patients distributed throughout the United States provide a remarkable window into the oral health status of a diverse patient population. BigMouth has been used to support, or refute, hypotheses of research proposals by generating preliminary data for funding applications and has therefore influenced the direction of oral health research. Moreover, BigMouth has been used to assess the quality of health intervention for specific patient populations, for example, providing dental care to women while pregnant,^11^ patients with diabetes,^12^ or children receiving sealants.^13^^,^^14^ Importantly, to date, the 11 participating BigMouth institutions are all academic—but vary in clinic size and resources for conducting research, and the results generated by BigMouth studies have started to illustrate that dental treatments in these educational settings are safe, effective, and cost-efficient.^15^^,^^16^

### Research education mission

BigMouth provides a secure environment in which oral health researchers at all levels (faculty, dental students, specialty trainees, master-level students, and doctoral-level students) have rapid access to a large dataset for analysis. Crucially, BigMouth serves as a “sandbox” to learn how to conduct clinical research using “real-world data,” appreciating all their limitations, including data availability, data quality, and challenges of electronic phenotyping.^17^

### Research priorities

By pooling datasets, BigMouth presents a more complete picture of types of patients. More specifically, BigMouth facilitates the study of oral manifestations of rare diseases that can lead to better understanding of the factors that affect more common diseases. Such rare diseases have a prevalence of fewer than 5 cases per 10 000 population^18^ with a 15% manifestation with oral-facial symptoms. As it takes on average 7 years to diagnose a rare disease, dentists can significantly influence identifying a rare disease by its oral symptomatology and help manage oral and overall quality of life.^19^ Infrequent diseases with oral health syndromes^20^ may be easier to locate in dental EHRs; however, individual institutions may not have enough patients with the disease for meaningful research. Another critical priority of BigMouth is the connection of oral health with general health. Information exchange between medical and dental EHRs is sorely limited because of ongoing interoperability issues, lack of consensus of what key components should be recorded in a patient’s record, and lack of documentation standards for dental EHRs.^21^^,^^22^ BigMouth includes medical history data and data on medications, as such allowing for investigation of the oral health-overall health relationship.^23^ Importantly, BigMouth will also allow for the building of a sustainable dental learning health system focused on providing patients with safe and effective oral health care.

## GOVERNANCE

As part of the formation of BigMouth, a data governance framework was developed that encouraged data sharing while allowing control of contributed data.^9^ BigMouth was originally conceived and remains as a single central repository, where all sites deposit their data. While there have been discussions about moving to a federated model, the complexity of requiring contributing sites to host their own i2b2 instances was a major barrier. Over time, changes were made to encourage site participation, access of data, execution of research studies, and sustainability. Specifically, data from beyond the original dental EHR (axiUm, Vancouver, Canada) were accepted by BigMouth, allowing for more sites to participate. This is important as several institutions are moving to Epic (Epic, Verona, WI). The Governance Committee permits noncontributing sites to receive data from BigMouth as long as those queries are for noncommercial purposes. The decision to limit access to data in BigMouth to nonprofits was based on advice from institutional legal representatives to ensure there was no perception that patient data were being monetized. As our institutions are gaining experience and developing formal policies for collaborating with for-profit entities for discovery using EHR data, we expect to revisit our restrictions on limiting access.

A 3-year National Library of Medicine resource development grant (G08LM010075) originally supported the formation of BigMouth and sharing of data from the 4 founding institutions. Supporting sustainability, a one-time setup fee of $10 000 has been implemented for new sites, and all sites pay an annual fee of $2500 which supports updating the repository on a quarterly basis. Although there is no cost for participating sites to query summary data using i2b2 web interface, there is now a fee schedule, based on complexity, for requests that require extracting data with costs for noncontributing members being higher than for contributors.

## OPERATIONS

### Conducting research using BigMouth: approaches and lessons learned

Researchers aspiring to use BigMouth data submit a proposal using a predefined template. Each contributing site has a representative who will first independently review the proposal based on scientific merit, potential overlap with other approved BigMouth projects, and if the institution agrees to share data for the proposed research. To date, the BigMouth committee has formally reviewed and approved 18 research proposals. In order to minimize the back and forth, researchers are now invited to join the committee discussion which has led to a robust process with faster approvals.

BigMouth has been used for a variety of scientific purposes, including:

1. Clinical Research: For example, assessing the use of opioid and antibiotics medications in academic dental settings.
2. Quality Improvement: As BigMouth contains structured data, it is particularly amenable for quality measurement.^11^^,^^12^^,^^24^
3. Operations: For example, enhancement of the Odontosearch tool to help identify human remains.^25^
4. Educational Research: For example, assessing the value of a generalist versus specialist teaching model for periodontics.^26^

BigMouth is emerging as an indispensable tool that has served as a data source for our learners^27^ and faculty.^9^^,^^28–30^

Challenges of using BigMouth match those reported for the use of EHR data for research. EHR data are primarily collected for clinical purposes, and are not entirely representative of the population, contain missing data, may imperfectly characterize outcomes, have uncalibrated clinicians input data, and are likely to contain various levels of accuracy.^31^ Through COHRI, the contributing sites are encouraged to use standardized data collection tools such as a dental diagnostic terminology (SNODDS) and medical and dental history data collection forms. These standardized terminologies have formed the basis for allowing users to query BigMouth. Each user logging into BigMouth can view 2 folders in the ontology (a) site-level terminology and (b) COHRI terminology. A site-level terminology contains terms from the local EHR as is without many transformations. This hierarchy provides users an opportunity to browse through terms that they are familiar with and run queries to get patient counts at their local institution. The BigMouth common data model or “COHRI” terminology combines concepts from all institutions and allows users to run queries across the entire database.

Data accuracy is often difficult to determine, as there are no external data sources for validation purposes, and is often ascertained by assessing if these data are within expected boundaries. Patients also do not always report medical comorbidities such as diabetes and hypertension status to dentists, leading to possible underreporting in the dental EHR.^32^ Assessing the consistency of the data is even more challenging as the data come from various institutions with a mix of learners and faculty providers.

The BigMouth technical team and researchers work together during the data extraction phase to identify data quality issues. Data quality is checked after data are extracted from sites, and after the load process. Quality checks after data extraction are conducted through an automated script which compares data received from all institutions with the previous extract received from the same site to flag any possible issues. Quality checks are also performed after data are loaded though a system sanity checklist that is used as a guideline to test both data and the functionality of the BigMouth querying interface. There is also often a virtuous cycle where any data quality issues can be communicated to the contributing sites, who can make changes to their EHR to mitigate concerns for the future. We have also found that clinical users, who are often reticent in having to collect structured data in the EHR while treating patients, become more understanding of the importance of secondary data use.

Lastly, we have found the need to provide training sessions that cover the use of the i2b2 web interface in order to explore the type and amount of data available, appropriate observational study designs that can be used, how to formulate research questions, and how to submit a full project proposal.

### Ongoing adoption of BigMouth

Contributing institutions value their inclusion in BigMouth as they gain access to a large national dental dataset which directly or indirectly has fostered new research collaborations, provided diverse clinical, epidemiological, statistical, and informatics expertise, and provided an additional venue for professional development. Barriers for other institutions to become a BigMouth contributor include limited technical personnel supporting the data extraction process, costs, lack of perceived value, and absence of leadership support.

New users who are interested in contributing data must be members of COHRI whose mission is to promote and support collaboration for research and education amongst dental institutions.^10^ There continues to be excellent communication and overlap between the leadership of COHRI and members of the BigMouth project review committee. While our focus has been on onboarding new sites that use the axiUm EHR, we have recently pivoted to onboarding sites with different EHRs. New sites often join due to advocacy of their faculty who may have heard about BigMouth from other colleagues. We, therefore, anticipate that an institution’s decision to adopt BigMouth is more akin to a “complex contagion” as defined by Centola, where interest and adoption are driven by reinforcement from multiple sources or wide bridges.^33^

### Envisioning the future of BigMouth

We consider the 10-year development horizon for BigMouth to drive strategic developments and enable the broader community of data repository experts and learning health system advocates to forge collaborations that will widen the impact of data to improve health outcomes. Accordingly, we have 3 focus areas:

1. **Connecting with medical data:** The FDI World Dental Federation’s (FDI) definition of oral health reads: “Oral health is multifaceted and includes the ability to speak, smile, smell, taste, touch, chew, swallow and convey a range of emotions through facial expressions with confidence and without pain, discomfort and disease of the craniofacial complex.”^34^ Connecting a dental RPDR to a medical EHR might allow us to find correlations between periodontitis and cardiovascular disease.^35^^,^^36^
2. **Expanding types of data in BigMouth:** Our vision is to incorporate dental imaging data (2d and 3d) and mandate the use of standardized diagnostic terminologies by all contributing institutions. Connecting to mobile health apps that collect patient-reported outcomes^37–39^ as well as patient-reported experience measures^40^ is also crucial aspiring toward a more holistic definition of oral health.
3. **Using BigMouth to improve oral health care:** Dentistry lags behind the medical profession in fostering the uptake of research-informed treatments.^41–43^ The use of computerized knowledge management, for example, in the form of audit and feedback and clinical decision support has been introduced in the oral health arena^44–46^ and will undoubtedly facilitate alignment of every day dental practice with evidence-based guidelines.^47^ Hence, BigMouth is positioned to play an important role in getting dental teams and clinics to adopt and consistently use evidence-based oral health guidelines and will be a catalyst for the transition from payment-focused care^48^ to culturally sensitive, effective, and high-value oral health care.^14^

We have many challenges ahead, including the expansion of BigMouth to other contributing institutions. While we have developed a process for rapidly incorporating data from the axiUm, EHR, we will need to develop scalable approaches for incorporating data from other platforms. We also strive to include contributing institutions globally, which will require an understanding of legal and policy issues of sharing patient data across borders. While dental institutions have been willing to share patient data, we expect more challenges for connecting or incorporating data from the patient’s medical record. Returning to the FDI definition, we aspire to embrace this holistic definition and finally help move dentistry from treating disease to treating a person with disease.

## CONCLUSION

Visionary leadership, combined with a strong governance approach to data sharing, has made the large-scale dental data repository, BigMouth, a reality. Initial federal funding and ongoing efforts to develop sustainability have supported researchers’ efforts to mine data otherwise not available to advance dental research.

## FUNDING 

This work was supported initially by the National Library of Medicine (grant number G08LM010075).

## AUTHOR CONTRIBUTIONS

MFW, EK, and HS conceptualized the manuscript and wrote an initial draft. All authors expanded, proofread, and substantially edited the manuscript. MFW, EK, and JMW secured funding for the project.
